# Cu-MOF derived Cu–C nanocomposites towards high performance electrochemical supercapacitors

**DOI:** 10.1039/c9ra09738d

**Published:** 2020-01-28

**Authors:** Jun Wang, Mumin Rao, Changchun Ye, Yongcai Qiu, Wenjun Su, Sheng-run Zheng, Jun Fan, Song-liang Cai, Wei-Guang Zhang

**Affiliations:** School of Chemistry, South China Normal University Guangzhou 510006 China wgzhang@scnu.edu.cn; School of Environment and Energy, South China University of Technology Guangzhou 51006 China yeccstu@163.com; State Key Laboratory of Luminescent Materials and Devices, South China University of Technology Guangzhou China; Zhongshan Polytechnic Zhongshan Guangdong 528404 China; Guangdong Energy Group Science and Technology Research Institute Co., Ltd Guangzhou China; Key Laboratory of Education Ministry for Modern Design and Rotor-Bearing System, Xi'an Jiaotong University Xi'an 710049 China

## Abstract

For the development of asymmetric supercapacitors with higher energy density, the study of new electrode materials with high capacitance is a priority. Herein, the electrochemical behavior of nano copper in alkaline electrolyte is first discovered. It is found that there are two obvious reversible redox symmetric peaks in the range of −0.8–0.2 V in the alkaline electrolyte, corresponding to the conversion of copper into cuprous ions, and then converting cuprous ions into copper ions, indicating that the nanocomposite electrode has the characteristics of a pseudocapacitive reaction. It has a specific capacitance of up to 318 F g^−1^ at a current density of 1 A g^−1^, which remains at nearly 100% after 10 000 cycles at the same current density. When assembled with a Ni(OH)_2_-based electrode into an asymmetric supercapacitor, the device shows excellent capacitive behavior and good reaction reversibility. At 0.4 A g^−1^, the supercapacitor delivers a reversible capacity of 8.33 F g^−1^ with an energy density of 13.5 mW h g^−1^. This study first discovers the electrochemical behavior of nano copper, which can provide a new research idea for further expanding the negative electrodes of supercapacitors with higher energy density.

## Abstract 2

A new Cu–C nanocomposite derived from Cu-based metal–organic framework exhibits greatly improved electrochemical performance.

## Graphical abstract

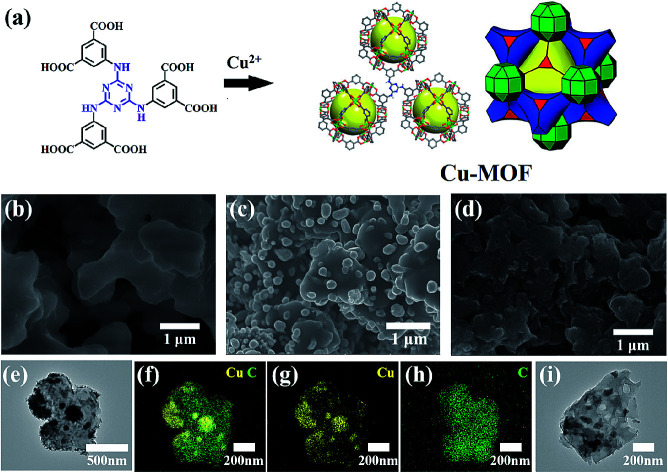

## 1. Introduction

As a new type of energy storage equipment between traditional capacitors and batteries, supercapacitors have the advantages of high power density, fast charge and discharge, long cycle time and low environmental pollution, thus they have wide application in the fields of spare power systems, portable electronic equipment, information technology and hybrid electric vehicles.^1–13^ However, the low energy density and multiplier performance of supercapacitors restrict their practical application. Therefore, it is important to develop new electrode materials with high electrochemical performance. Supercapacitors can be divided into symmetric supercapacitors and asymmetric supercapacitors. Compared with symmetric supercapacitors, asymmetric supercapacitors are widely considered because of their larger voltage window and higher energy density.

In asymmetric supercapacitors, transition metal compounds with high reoxidation–reduction potential such as metal oxides and sulphides, phosphates are usually used in the cathode. However, there are fewer types of anodes, and the usual reports are porous carbons and ferric oxide.^14–28^ Ferric oxide usually has poor electrical conductivity and needs to be combined with carbon materials, leading to reduction of its capacitance. Therefore, it is challenging to develop new anode materials with good conductivity and high capacitance.

Metal–organic frameworks (MOFs) are a kind of organic–inorganic hybrid material, which is composed of a metal center/cluster and organic ligands and constructed by self-assembly with one-dimensional, two-dimensional or three-dimensional porous crystal structures. Due to the diversity of organic ligands, MOFs are characterized by strong chemical modification and adjustable size, which achieves extensive potential applications in fluorescence, sensing, gas adsorption and separation, catalysis and other fields.^29–39^ In recent years, materials prepared by calcining MOFs as templates or precursors under different conditions, such as MOF based multi-porous carbon materials, metal oxides and their complexes, have shown excellent performance in lithium ion batteries, supercapacitors and other fields.^40–45^

Based on the above considerations, herein, the Cu–C nanocomposites derived from Cu-MOFs is prepared and fully characterized, where the copper nanoparticles are evenly dispersed in porous carbons. It is found that there are two obvious reversible redox symmetric peaks in the range of −0.8–0.2 V in alkaline electrolyte, corresponding to the conversion of copper into cuprous ions, and then to copper ions, indicating that the nanocomposite electrode has the characteristics of pseudocapacitive reaction. It delivers a specific capacitance of up to 318 F g^−1^, which remains nearly 100% after 10 000 cycles at a current density of 1 A g^−1^. This is the first time to discover the electrochemical behavior of nano copper, which can provide a new research interest for further expanding the anodes of supercapacitors with higher capacitance.

## 2. Materials and methods

### 2.1. Preparation of ligand

The ligand was synthesized according to the method described in the ref. 46. 13.5 g 5-aminophthalic acid, 10 g sodium hydroxide, 5 g sodium bicarbonate and 150 mL deionized water are stirred at room temperature for 30 min to prepared solution A. Then, 5 g of melamine was dissolved in 50 mL 1,4-dioxane, which was added to solution A. Then the mixture was transfered to a 500 mL round-bottomed flask and reflux at 100 °C for 24 h. After cooling to room temperature, hydrochloric acid was used to adjust the pH to acidity, and the ligand H_6_TDPAT was obtained by filtration, washing and drying.

### 2.2. Preparation of Cu–C nanocomposite

Cu(NO_3_)_2_·3H_2_O (2.416 g, 10 mmol) and H_6_TDPAT(1.236 g, 2 mmol) were dissolved in 150 mL DMF, and green powder was obtained by constant temperature reaction at 100 °C for 24 h in 200 mL reactor, namely Cu-MOF. The as-synthesized Cu-MOF was heated at 700 °C under argon atmosphere for 2 h to obtain the Cu–C nanocomposite.

### 2.3. Preparation of MOFs derived carbon

100 mL of 1 M FeCl_3_ and 1 M HCl aqueous solution was prepared. And then the as-synthesized Cu–C nanocomposite was added to the solution and then reflex at 80 °C for 12 h to remove Cu nanoparticles and obtain the porous carbon material.

### 2.4. Electrochemical measurements

The electrochemical properties of all samples were tested by CHI 760C electrochemical workstation (Shanghai chenhua), using conventional three-electrode system and two-electrode system, Pt as the counter electrode, Ag/AgCl as the reference electrode, and Cu-MOF or the derivatives as the working electrode. For the preparation of working electrode, the carbon paper was firstly ultrasonic cleaned in water and ethanol for 30 min, then was cut to size of 1 × 2 cm^2^ sheet. The materials, acetylene black and poly vinylidene fluoride were mixed with the ratio of 80 : 10 : 10 and then the solvent of *N*-methyl-2-pyrrolidone was added to prepare the slurry, which was coated on the carbon paper with the area of about 1 cm^2^ and then placed in oven at 120 °C for 5 h. The active material quality of each electrode piece was accurately weighed, and the electrode piece was prepared. Electrochemical performance test: 1 M KOH as electrolyte, Hg/HgO as reference electrode, electrode materials on carbon paper as working electrode, the electrochemical properties of the material was tested with three electrodes system, including cyclic voltammetric curves (CV), charge–discharge test (GCD), cycle stability test, supercapacitor device testing.

### 2.5. Characterization

The X-ray powder diffraction of the samples were measured on a Bruker D8 Advance diffractometer at 40 kV and 40 mA with a Cu target tube and a graphite monochromator. The specific surface area and pore-size distribution were obtained by N_2_ adsorption/desorption isotherm (Belsorp-max). The structure and morphology were characterized by field-emission scanning electron microscopy (SEM) (TESCAN Brno, s. r. o. MAIA3) and Transmission electron microscopy (TEM) (JEOL 2100F).

## 3. Results and discussion

**Fig. 1:**
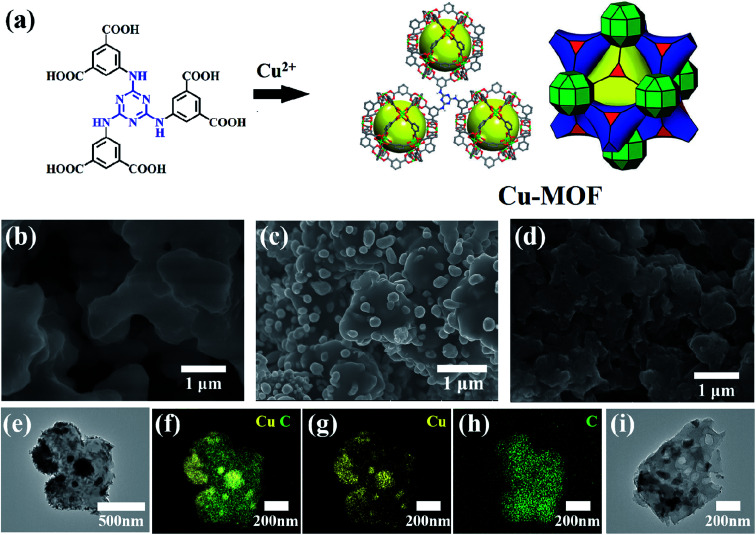
(a) The synthesis of Cu-MOF. SEM images of (b) Cu-MOF, (c) Cu–C and (d) carbon (C). (e) A typical TEM image of Cu–C nanocomposite. (f–h) The EDX mapping of Cu–C. (i) A typical TEM image of porous C obtained from removing Cu nanoparticles.

As shown in Fig. 1a, the ligand of H_6_TDPAT and Cu(NO_3_)_2_ can be synthesized into green powders by solvent method. Fig. 1b shows that the Cu-MOF are irregular nanoparticles with 1 micron in size by SEM characterization. As shown in Fig. 1c, the Cu–C derived from Cu-MOF are also irregular nanoparticles, and there are many small nanoparticles evenly distributed on them. After the Cu–C nanocomposite was soaked with Fe^3+^ to remove the elemental copper, the small particles on the surface of the nanoparticles disappeared, indicating that the small particles on the surface of the Cu–C nanocomposite should be copper. Fig. 1e shows a typical TEM image of the Cu–C nanocomposite, the Cu nanoparticles are evenly distributed in the porous carbon, which can be further proved by the EDX mapping (Fig. 1f–h). The TEM of the carbon (C) after removing the Cu nanoparticles also is display in Fig. 1i.

**Fig. 2:**
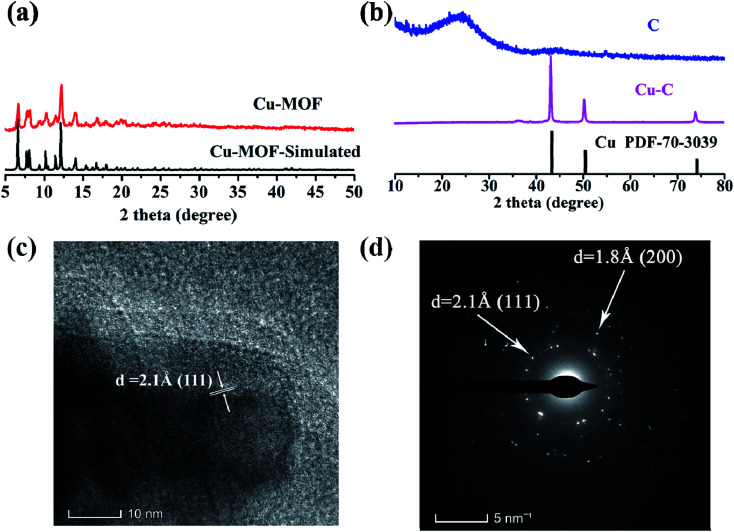
The XRD patterns of (a) Cu-MOF and (b) Cu–C and C. (c) The HRTEM of Cu–C. (d) The SAED map of Cu–C.

As shown in Fig. 2a, it is found that the as-synthesized Cu-MOF well matches the simulated XRD pattern, indicating the purity of the Cu-MOF. By carbonizing Cu-MOF at 700 °C, the corresponding materials can be obtained. XRD tests show that the material is a Cu–C nanocomposite. As shown in Fig. 2b, for the Cu–C nanocomposite, the peaks at 43.3°, 50.4° and 74.1° correspond to the crystal surfaces (111), (200) and (220) of Cu, respectively, indicating the existence of Cu. The HRTEM (Fig. 2c) shows lattice fringes with inter plane spacing of 0.21 nm, corresponding to the (111) plan of Cu. The bright rings in selective area electron diffraction (SAED) pattern (Fig. 2d) reveal interplanar distances of 0.18 nm and 0.21 nm corresponding to the (200) and (111) plane of Cu. These results further prove the presence of simple copper in Cu–C and its good crystallinity. When the Cu–C nanocomposite was soaked in Fe^3+^/HCl, it can be seen that the characteristic peak of elemental Cu was disappeared, leaving only a broad peak of carbon around 23 °C, indicating that Cu was removed from Cu–C to obtain the carbon (Fig. 2b).

**Fig. 3:**
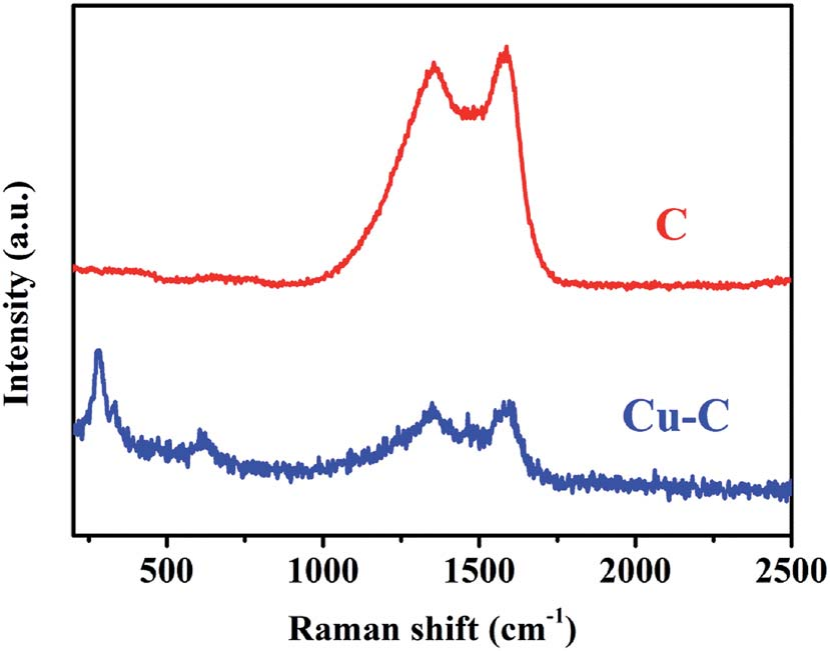
The Raman spectra of Cu–C and C.

**Fig. 4:**
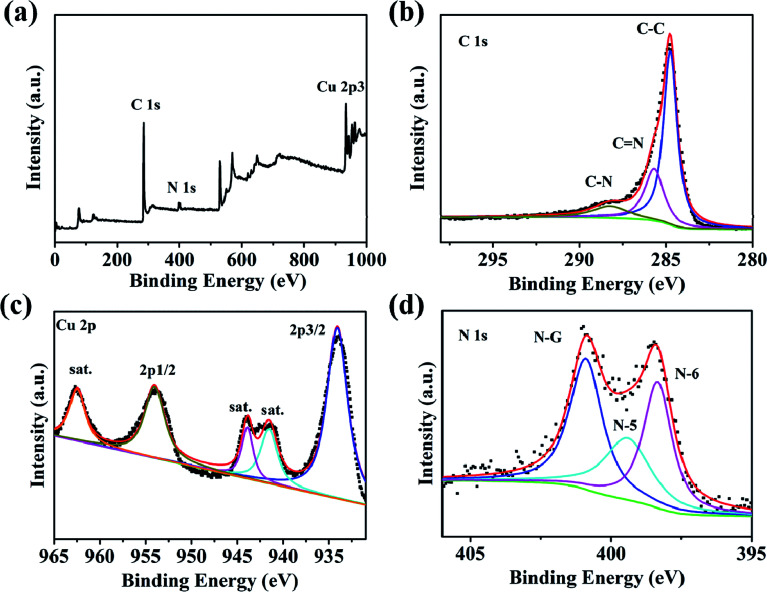
(a) XPS survey spectrum of Cu–C. High-resolution XPS spectra for (b) C 1s, (c) Cu 2p and (d) N 1s.

Through the analysis of the Raman spectra Cu–C and C, it is found that the Cu–C nanocomposite not only has D band and G band of carbon material, corresponding to the peaks of 1352 cm^−1^ and 1588 cm^−1^, respectively, but also has the peaks of Cu at about 280.7 and 619.6 cm^−1^. However, after removing Cu, only Raman peak of carbon material exists (Fig. 3). The surface elemental configuration and chemical bonding states of the Cu–C nanocomposite were characterized by XPS. As shown in Fig. 4a, the complete spectrum shows that the existence of Cu, C, N elements in Cu–C, which also was confirmed by the EDS mapping (Fig. 1f and g). Fig. 4b presents the C 1s XPS spectrum. The binding energies of 285.7 eV and 288.3 eV correspond to the C=N and C–N, respectively. The Cu 2p XPS spectrum presented in Fig. 4c shows that there are two peaks at 953.9 eV and 934.1 eV, corresponding to the Cu 2p_1/2_ and Cu 2p_3/2_, respectively, confirming the existence of Cu in the sample. Besides, the N 1s XPS spectrum in Fig. 4d shows that the nitrogen element also exists in the Cu–C sample, which are 400.9 eV, 399.4 eV and 398.3 eV, corresponding to the graphite nitrogen, pyrrole nitrogen and pyridine nitrogen, respectively.

**Fig. 5:**
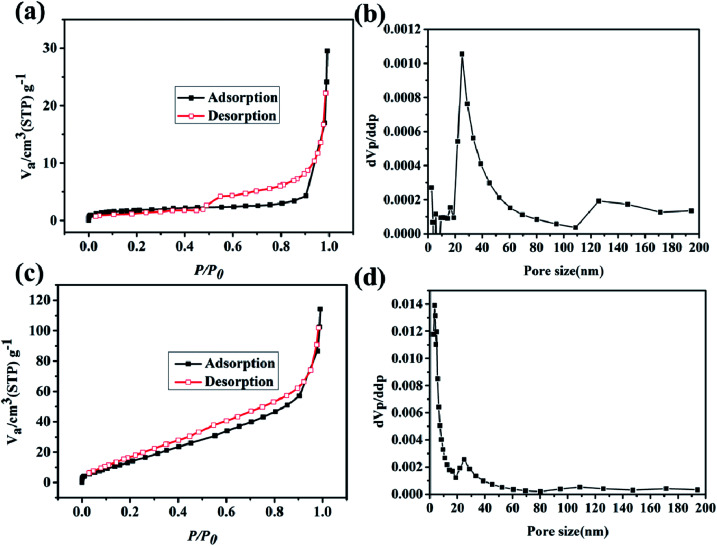
The N_2_ adsorption/desorption isotherms of (a) Cu–C and (c) C. The pore size distributions of (b) Cu–C and (d) C.

To further test the BET and pore size distribution of the materials, the nitrogen adsorption test was conducted at 77 K. As shown in Fig. 5a, the N_2_ adsorption curve of the Cu–C nanocomposite presents a type-II adsorption curve with a specific surface area of 6.4 m^2^ g^−1^. The pore side distribution in Fig. 5b shows that the pore size of the Cu–C nanocomposite is mainly distributed around 30 nm, indicating that the Cu–C nanocomposite is mesoporous. When Cu-MOF is carbonized, copper ions in MOFs may first form copper oxide, and then undergo reduction reaction with carbon materials at high temperature, consuming part of carbon and then forming Cu–C complex with mesoporous pore size. When using hydrochloric acid to remove the copper in the Cu–C nanocomposite, the porous carbon can be obtained. As shown in Fig. 5c, its nitrogen adsorption curve is also a type-II feature, indicating the mesoporous nature. Its specific surface area is 72.7 m^2^ g^−1^, which is much higher than the Cu–C nanocomposite. The reason is that when Cu is removed by acid, the carbon material has more pore structure due to the removal of copper, so that its specific surface area increases, and the pores with about 5 nm appear.

**Fig. 6:**
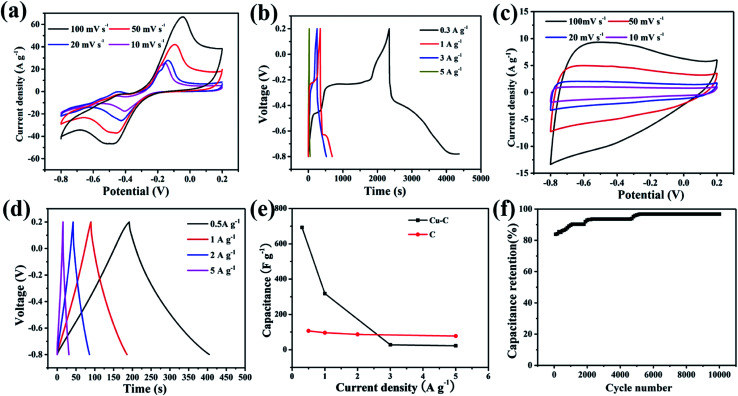
(a) CV curves for Cu–C nanocomposite electrode, (b) GCD curves of Cu–C nanocomposite electrode, (c) CV curves for C nanocomposite electrode, (d) GCD curves of C nanocomposite electrode, (e) comparison of specific capacity at different current densities, (f) electrochemical stability of the Cu–C electrode at current density of 1 A g^−1^ over 10 000 cycles.

In order to study the electrochemical performances, the Cu–C nanocomposite was fabricated into working electrodes and the electrochemical behaviors were measured at the selected charge and discharge electrode potential interval. 1 mol L^−1^ KOH was selected as the electrolyte and tested through a three-electrode system. The voltage window was set between −0.8–0.2 V, and the scanning speed was 10, 20, 50, and 100 mV s^−1^. The CV curve showed obvious redox symmetric peaks, indicating that the Cu–C nanocomposite electrode has the characteristic of pseudocapacitive reaction (Fig. 6a). In addition, the charging and discharging performance of the Cu–C nanocomposite was tested with the current densities of 0.1, 3, 5, 7 and 10 A g^−1^. According to the galvanostatic charge/discharge (GCD) curve shown in Fig. 6b, the GCD curve of Cu–C nanocomposite shows obvious nonlinear characteristics. It is noted that there are two plateaus in the charging and discharging process with good symmetry. The CV and GCD curves of the Cu–C nanocomposite both indicate that two oxidation reactions and two reduction reactions have taken place in the alkaline electrolyte. The redox process can be described as following: the elemental copper is first converted into cuprous ion and the CuOH was formed (eqn (1)), and then into copper ion to form Cu(OH)_2_ (eqn (2)). The whole process has good reversibility. These is an impressive result because the appearance of the plateaus will greatly increase the capacitance, which means that the Cu–C nanocomposite may be a promising anode for high performance supercapacitors. After calculation, it is concluded that the specific capacitance of the Cu–C nanocomposite is 693 F g^−1^, 318 F g^−1^, 28 F g^−1^ and 22 F g^−1^ when the current density is 0.3 A g^−1^, 1 A g^−1^, 3 A g^−1^ and 5 A g^−1^, respectively. As a template for Cu–C materials, MOFs enables nanometer copper to be dispersed well on carbon materials, which can avoid agglomeration of Cu and improve the performance of supercapacitors. Compared with the reported anodes such as porous carbons, as shown in Table 1, the Cu–C has the considerable capacity of up to 318 F g^−1^ at a current density of 1 A g^−1^. The experimental results show that the Cu–C nanocomposite is a good electrode material under the alkaline electrolyte environment.

1 Cu + OH^−^ ↔ CuOH + e^−^

2 CuOH + OH^−^ ↔ Cu(OH)_2_ + e^−^

**Table 1:** Summarized properties of our and recently reported anodes

Materials	Capacity (F g^−1^)	Current density (A g^−1^)	Ref.
NMHCSs	240	0.2	47
CSiO_2_	107.9	0.5	48
NHPCNF	394	1	49
N-CNTs	63	0.1	50
NO-PC	269	1	51
PANI@CNT-CNC/PVA-PAA	164.6	1	52
**Cu–C**	**318**	**1**	**This work**

In order to further study the role of Cu in the Cu–C nanocomposite, the electrode performance of carbon material also was tested at the same conditions. As shown in Fig. 6c and d, the CV curves and the GCD curves of porous C are different. The CV curves of porous C change into a rectangular shape with the characteristics of the electric double layer capacitors, illustrating that the removal of nanometer copper has also removed the pseudocapacitance of the material. The GCD curves of C present an isosceles triangle, with a linear relationship between time and potential. The charging curve and discharge curve are very symmetric, showing that the porous carbon nanocomposite material has good reversibility. By calculation, it is concluded that when the current density is 1 A g^−1^, the specific capacity is 96 F g^−1^, which is much lower that of the Cu–C nanocomposite. By comparison, nano-copper thus plays a very significant role in the increase of electrochemical capacitance. The nano Cu is the active material in the Cu–C nanocomposite.

Fig. 6e shows the changes of specific capacitances with the current densities calculated by the integral area of curves by cyclic voltammetry. With the increase of current density, the specific capacitance of the Cu–C nanocomposite has a significant trend of decline, while the specific capacitance of the porous C is less affected by the change of current density. With the increase of current density, diffusion limits the movement of electrolyte ions, which will generally lead to a decrease in the utilization rate of materials. Porous carbon stores charge through the surface, while the redox reaction of the Cu–C nanocomposite, especially the elemental Cu cannot be completely reacted with the increase of current density. Fig. 6f shows the cyclic stability of the Cu–C nanocomposite under 10 mV s^−1^ sweep speed curve. It can be seen that the capacitance shows linear increase and then levels off. The phenomenon can be due to the continuous activation of nano-copper till reaction completion.

**Fig. 7:**
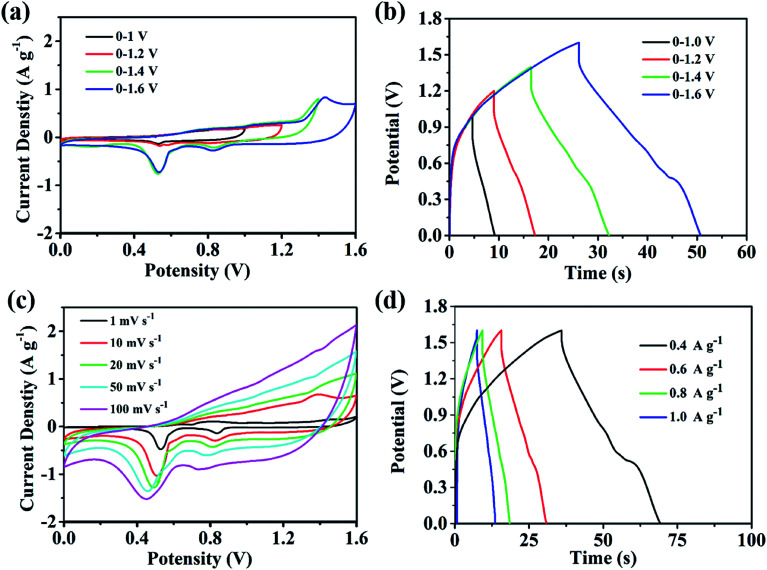
The performance of the supercapacitor (a) cyclic voltammetry curve of different voltage windows (sweep speed of 100 mV s^−1^); (b) charging and discharge curves for different voltage windows (current density of 0.4 A g^−1^); (c) a series of cyclic voltammetric curves at different speeds; (d) a series of charging and discharging curves at different speeds.

**Fig. 8:**
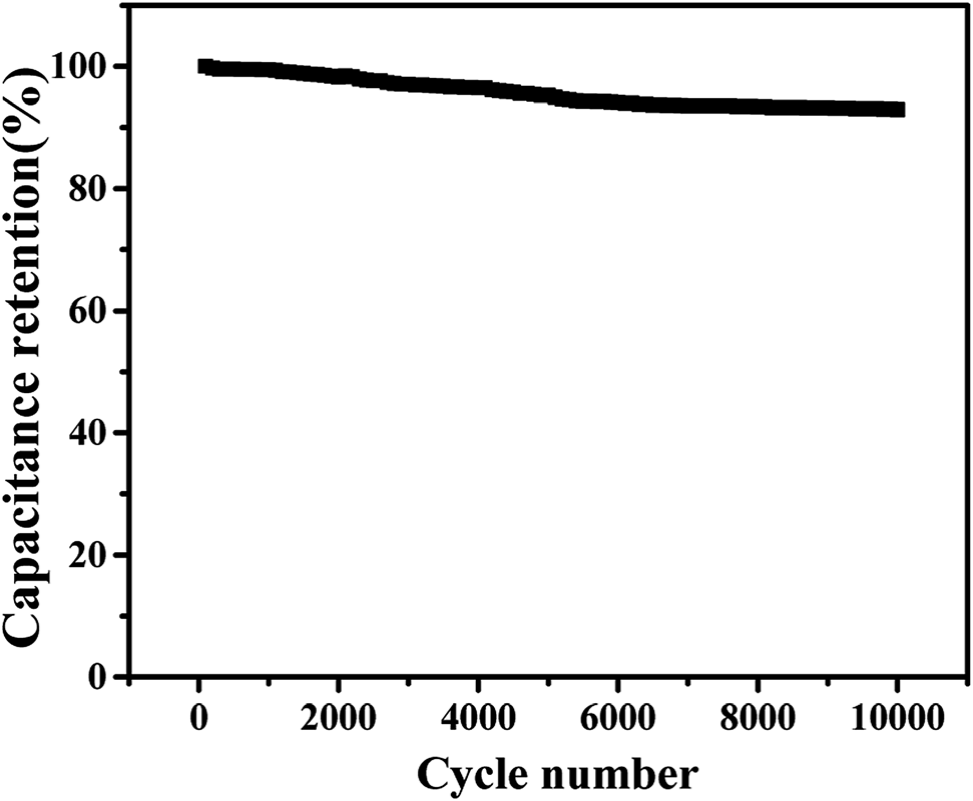
The cyclic stability of the supercapacitor over 10 000 cycles at a current density of 0.4 A g^−1^.

The assembly of high-performance supercapacitors should consider the balance of positive and negative electrodes. In the assembly process of our supercapacitors, the Cu–C nanocomposite as the negative electrode and commercial Ni(OH)_2_ as the positive electrode are used to assemble the asymmetric supercapacitors with 1 M KOH as the electrolyte. In order to study the maximum working voltage of the devices, the cyclic voltammetric of different voltage range (scanning speed of 100 mV s^−1^) and charge–discharge performance (current density of 0.4 A g^−1^) were tested. Fig. 7a shows the cyclic voltammetric curves with the voltage window from 1.0 to 1.6 V. It can be found that there is no obvious change for the curve shape, suggesting that the supercapacitor can be even extended to 1.6 V voltage window. At the current density of 0.4 A g^−1^, when the operating voltage window of the device increases from 1.0 V to 1.6 V, the charge and discharge curves measured by the supercapacitor under different voltage windows show nonlinear symmetry. As we all know, a large voltage window is also conducive to the improvement of energy density. Therefore, we will select the voltage window of 0–1.6 V to further study the electrochemical properties of the device. As shown in Fig. 7c, when the voltage window is 0–1.6 V, the cyclic voltammetry curve of the device in the sweep range of 1–100 mV s^−1^ shows good consistency, indicating that it has ideal capacitive behaviour and rate performance. The Fig. 7d shows the constant-current charge and discharge test results of the device within the current density range of 0.4–1 A g^−1^. The charge and discharge curves can maintain good symmetry, and the device has excellent capacitive behaviour and good reaction reversibility. At 0.4 A g^−1^, the supercapacitor delivers a capacity of 8.33 F g^−1^ and an energy density of 13.5 mW h g^−1^. In addition, we also tested the stability of the assembled supercapacitor. As shown in Fig. 8, before 5000 cycles, the capacitance dropped slightly, but still reached over 90%, showing that the asymmetric supercapacitor has good electrochemical stability.

## 4. Conclusion

In summary, the electrochemical behavior of nano copper in alkaline electrolyte was investigated in this work for the first time. It is found that there are two obvious reversible redox symmetric peaks in the range of −0.8–0.2 V in the alkaline electrolyte, corresponding to the conversion of copper into cuprous ions, and then to copper ions, indicating that the nanocomposite electrode has the characteristics of pseudocapacitive reaction. It has a specific capacitance of up to 318 F g^−1^ at the current density of 0.1 A g^−1^, which remains nearly 100% after 10 000 cycles. This is the first time to discover the electrochemical behavior of nano copper, which provides a new research idea for further expanding the anode electrode of supercapacitor with higher capacitance.

## Conflicts of interest

There are no conflicts to declare.

## Supplementary Material
